# The Peripheral Binding of 14-3-3γ to Membranes Involves Isoform-Specific Histidine Residues

**DOI:** 10.1371/journal.pone.0049671

**Published:** 2012-11-26

**Authors:** Helene J. Bustad, Lars Skjaerven, Ming Ying, Øyvind Halskau, Anne Baumann, David Rodriguez-Larrea, Miguel Costas, Jarl Underhaug, Jose M. Sanchez-Ruiz, Aurora Martinez

**Affiliations:** 1 Department of Biomedicine, University of Bergen, Bergen, Norway; 2 Facultad de Ciencias, Departamento de Quimica Fisica, Universidad de Granada, Granada, Spain; 3 Department of Chemistry, University of Oxford, Oxford, United Kingdom; 4 Laboratorio de Biofisicoquímica, Departamento de Fisicoquímica, Facultad de Química, Universidad Nacional Autónoma de México, México DF, México; University of Queensland, Australia

## Abstract

Mammalian 14-3-3 protein scaffolds include seven conserved isoforms that bind numerous phosphorylated protein partners and regulate many cellular processes. Some 14-3-3-isoforms, notably γ, have elevated affinity for membranes, which might contribute to modulate the subcellular localization of the partners and substantiate the importance of investigating molecular mechanisms of membrane interaction. By applying surface plasmon resonance we here show that the binding to phospholipid bilayers is stimulated when 14-3-3γ is complexed with its partner, a peptide corresponding to the Ser19-phosphorylated N-terminal region of tyrosine hydroxylase. Moreover, membrane interaction is dependent on salts of kosmotropic ions, which also stabilize 14-3-3γ. Electrostatic analysis of available crystal structures of γ and of the non-membrane-binding ζ-isoform, complemented with molecular dynamics simulations, indicate that the electrostatic potential distribution of phosphopeptide-bound 14-3-3γ is optimal for interaction with the membrane through amphipathic helices at the N-terminal dimerization region. In addition, His158, and especially His195, both specific to 14-3-3γ and located at the convex lateral side, appeared to be pivotal for the ligand induced membrane interaction, as corroborated by site-directed mutagenesis. The participation of these histidine residues might be associated to their increased protonation upon membrane binding. Overall, these results reveal membrane-targeting motifs and give insights on mechanisms that furnish the 14-3-3γ scaffold with the capacity for tuned shuffling from soluble to membrane-bound states.

## Introduction

The 14-3-3 protein family is ubiquitously expressed in all eukaryotic cells and consists of seven isoforms in human, i.e. β, ε, η, γ, τ (also called θ), ζ and σ [1], [2], [3]. The 14-3-3 proteins are dimeric in their native state, with cuplike shaped monomers of ∼30 kDa with nine anti-parallel helices. These isoforms are highly conserved in sequence and structure both within the protein family in humans and across species, with highest conservation in the inner, concave surface, which is also the phosphopeptide-binding pocket [3], [4] (Figure S1). The 14-3-3s are scaffold proteins that primarily bind Ser/Thr phosphorylated peptides and proteins, but they also bind some non-phosphorylated motifs, such as WLDLE [5], as well as specific proteins such as SelW, which seems to regulate 14-3-3 in a redox-based mechanism [6], [7]. With more than 200 binding partners, 14-3-3 proteins are involved in nearly all important cellular processes, such as transcription, regulation of the cell cycle and metabolism, intracellular trafficking and targeting, cytoskeletal structure and apoptosis [8], [9], [10]. Hence, the physiological relevance and increased interest of these proteins as biomarkers and therapeutic targets is substantiated [11], [12].

As modulators of the subcellular location, the 14-3-3 proteins appear to play an important role in the promotion of cell surface expression of membrane proteins, notably by mechanisms that involve binding at specific signal motifs at the partner proteins [13], [14]. However, a possible contribution of 14-3-3 proteins to membrane targeting through direct interaction with the phospholipid bilayers has not been elucidated. 14-3-3 isoforms are actually found in the cytoplasm and often enhance phosphorylation-dependent cytoplasmic and/or intranuclear localization of their partners, as is the case for lipin-1 in adipocytes [15], BAD [16], and Skp2 [17]. However, there seems to be a certain 14-3-3 isoform specificity with respect to membrane affinity [18], [19], [20], [21]. Thus, 14-3-3γ and to a lesser extent 14-3-3ε are mostly localized in the cytoplasm, but the two isoforms also show affinity for natural chromaffin granule membranes and negatively charged phospholipid bilayers [20], [21], [22]. Moreover, 14-3-3γ colocalizes with the muscle-specific receptor tyrosine kinase at neuromuscular postsynaptic membranes [23]. We have recently shown that membrane binding most probably involves residues in an area different from the phosphopeptide-binding pocket of 14-3-3γ, and that the protein binds to membranes through peripheral interaction [21], [24]. Thus, the membrane interaction of 14-3-3γ resembles that of amphitropic proteins, which shuttle between a soluble state and a peripheral membrane-associated state in a controlled manner, and play important roles in transport, cell signaling, compartmentalization and cytoskeletal organization [25], [26], [27]. There are a number of systems where membrane interaction has been shown to be mediated by specific modular membrane-targeting domains [28] and different regulatory switches have been revealed as mechanisms for peripheral membrane-interaction in proteins, such as modulation of membrane lipid composition and/or modification of the protein by phosphorylation, myristoylation or prenylation, among others [27]. There are, however, a number of peripheral proteins where post-translational functional groups or specific domains are not implicated in the membrane interaction, which has been shown to be dependent on intrinsic properties of the polypeptide sequence and often involve helical anchors [26]. Little is known on the membrane-binding mechanism for 14-3-3γ, but it seems clear that the interaction does not require myristoyl or other lipid-related targeting-motifs [21].

In this work, we have investigated the membrane-interacting motifs of 14-3-3γ, aiming to contribute to the understanding of the binding mechanism at the molecular level. The influence of the ligand was first investigated using a Ser19-phosphorylated 43-residue-long polypeptide, i.e. THp-(1-43), corresponding to the N-terminal region of the isoform 1 of human tyrosine hydroxylase (TH). TH is the rate-limiting enzyme in the synthesis of catecholamines, and an established binding partner of 14-3-3 proteins [29]. Like Ser19-phosphorylated TH, the THp-(1-43) peptide is a high-affinity ligand of 14-3-3 proteins [21]. The interaction of 14-3-3γ with membranes was studied by surface plasmon resonance (SPR) using liposomes of defined phospholipid composition. These experiments have shown that the phosphopeptide stimulates the binding, and that salt is a necessary requirement for membrane interaction. By circular dichroism (CD) and differential scanning fluorimetry (DSF) it has also been demonstrated that salts of kosmotrope anions from the Hofmeister series (citrate^3−^, SO_4_^2−^, HPO_4_^2−^ and Cl^−^) bind 14-3-3γ and enhance the thermal stability of the protein. Molecular dynamics (MD) simulations and pH-dependent electrostatic analyses of 14-3-3γ and 14-3-3ζ, the latter used as a non-membrane binding counterpart, further support that phosphopeptide-bound 14-3-3γ might adopt an optimal charge distribution for interaction with the bilayer through the convex side of the N-terminal dimerization region of the protein. The lateral area surrounding His158 and His195 might also contribute with favorable longer-range electrostatic interactions in the consolidation and stabilization of membrane binding, and the involvement of these histidine residues in the association was proved by site-directed mutagenesis. Our results contribute to elucidate motifs and molecular mechanisms of membrane binding for 14-3-3γ. The peripheral membrane interaction of this protein seems modulated by conformational and electrostatic changes effected by phosphorylated ligand binding.

## Materials and Methods

### Materials

The peptides TH-(1-43), i.e. MPTPDATTPQAKGFRRAVSELDAKQAEAIMSPRFIGRRQSLIE, and its Ser19-phosphorylated counterpart THp-(1-43), i.e. MPTPDATTPQAKGFRRAVS(PO_3_)ELDAKQAEAIMSPRFIGRRQSLIE, were synthesized by CPC Scientific (San Jose, CA, USA) at approx. 90% purity, as seen by mass spectroscopy. The phospholipids phosphatidylcholine (PC) from egg yolk lecithin (95% PC) and porcine brain phosphatidylserine (PBPS) were purchased from Avanti Polar Lipids, Inc. (Alabaster, AL, USA). The absence of fatty acid oxidation in the mixtures was kindly verified by Sonnic Meier as reported [21]. Degassed and filtered ready to use HBS-P (10 mM Hepes, pH 7.4, 150 mM NaCl, 0.005% surfactant P20) and HBS-N (10 mM Na-Hepes, pH 7.4, 150 mM NaCl) buffers, as well as CM5 and L1 sensor chips were purchased from Biacore AB (GE Healthcare Bio-Sciences Ltd, Piscataway, NJ, USA).

### Site-directed mutagenesis

The missense mutations Y117F, H158F, H164E and H195S were introduced into 14-3-3γ cDNA using QuikChange® site-directed mutagenesis kit from Stratagene® (La Jolla, CA, USA). Mutagenesis was carried out using the following primers (from Sigma-Aldrich, St. Louis, MO, USA): Y117F forward, 5′-GCAGCGAGACCCAGT**TT**GAGAGCAAAGTGTTC and reverse, 5′-GAACACTTTGCTCTC**AA**ACTGGGTCTCGCTGC; H158F forward, 5′-GGCCTACAGCGAAGCC**TTT**GAGATCAGCAAAGAGC and reverse, 5′-GCTCTTTGCTGATCTC**AAA**GGCTTCGCTGTAGGCC; H164E forward, 5′-CGAGATCAGCAAAGAG**GAA**ATGCAGCCCACCCACC and reverse, 5′-GGTGGGTGGGCTGCAT**TTC**CTCTTTGCTGATCTCG; H195S forward, 5′-GAGCAAGCGTGC**AG**CTTGGCCAAGACC and reverse, 5′-GGTCTTGGCCAAG**CT**GCACGCTTGCTC. For the double mutant H158F/H195S the plasmid with the H195S mutation was subjected to mutagenesis with the H158F forward/reverse primers. The selected positive clones were verified by sequencing.

### Expression and purification of proteins, dialysis and buffer exchange

Wild-type (wt) and mutant human 14-3-3γ and 14-3-3ζ were expressed in *E. coli* (BL-21, codon+) as glutathione *S*-transferase fusion proteins, purified on glutathione-Sepharose 4B (GE Healthcare Bio-Sciences Ltd). Cleavage of the GST-14-3-3 fusion protein by thrombin and further purification of the isolated 14-3-3 proteins was performed as reported [21]. The purified proteins, in 50 mM Na-phosphate, pH 7.4, 150 mM NaCl, 1 mM dithiothreitol, 1 mM EDTA, were up-concentrated with filters of 30 kDa cut-off (Amicon ultracentrifugal filters, Millipore) and stored in liquid nitrogen until use. The dialysis of purified 14-3-3γ and 14-3-3ζ and buffer exchange to phosphate-free buffer, customarily 10 mM Na-Hepes, pH 7.4, was performed overnight at 4°C in Slide-A-Lyzer® Dialysis Cassettes from Pierce Chemical Company (Thermo Fisher Scientific Inc., Rockford, IL, USA) of molecular weight cut-off of 10 000. Buffer was changed twice during the process. The phosphate-free dialyzed proteins were referred to as d14-3-3γ and d14-3-3ζ.

### Preparation of liposomes

The procedures used for lipid handling and for preparation and characterization of large unilamellar vesicles (LUVs; from here referred to as liposomes) have recently been described in detail [21], [30]. Freshly made liposomes of neutral PC or of a combination of PC and negatively charged PBPS (PC∶PBPS; 1∶1) and with a size distribution of 105±25 nm were used [21].

### Surface plasmon resonance (SPR)

The SPR analyses were carried out with the Biacore 3000 biosensor (Biacore AB) at 25°C using the sensor chip CM5 with HBS-P as running buffer for monitoring the interaction of THp-(1-43) with 14-3-3 proteins, and sensor chip L1 with HBS-N buffer for the interaction of the 14-3-3 proteins with liposomes, essentially as described in [21], except as otherwise indicated. For the analysis of the effect of kosmotropic salts on the interaction of THp-(1-43) with 14-3-3γ, wt-d14-3-3γ was diluted in 10 mM sodium acetate, pH 4.5, and immobilized covalently to the hydrophilic carboxymethylated dextran matrix of CM5 by the standard primary amine coupling reaction, as described by the manufacturer, resulting in depositions of about 5 000 response units (RU). A reference surface was subjected to the same procedure but without protein. A stable base line was obtained in the cell with immobilized protein by a continuous flow (50 µl/min) of HBS-P as running buffer for about 1 h. The THp-(1-43) peptide was diluted to the indicated concentration with 10 mM Na-Hepes, pH 7.4, 0.005% P20, and the indicated salt additions, and injected over the immobilized d14-3-3γ in a volume of 100 µl for 3 min at a flow rate of 20 µl/min. For the analysis of membrane binding, liposomes made of either PC or PC∶PBPS were diluted with 10 mM Na-Hepes, pH 7.4, with the indicated salts, and injected at a flow rate of 10 µl/min, resulting in depositions of 4 000–6 000 RU. The protein samples were prepared at the indicated conditions, and injected over the immobilized liposomes at a flow rate of 10 µl/min. The sensor chip surface was regenerated by injecting isopropanol:50 mM NaOH (40∶60, v/v) and running buffer. The BIAevaluation program, version 3.2 (Biacore AB) was used for analysis of the sensorgrams.

### Circular dichroism (CD)

Far-UV CD measurements were performed with a Jasco J-810 spectropolarimeter equipped with a PTC-348WI Peltier element for temperature control at 25°C using a quartz cell with a path length of 1 mm. CD spectra of d14-3-3γ and 14-3-3γ, prepared in the indicated buffers and conditions and customarily at a concentration of 7 µM subunit, were acquired in the 200–260 nm range at a scan rate of 100 nm/min. 4 scans were averaged in each spectrum. Buffer scans were carried out at the same conditions and subtracted.

### Differential scanning fluorimetry (DSF)

We monitored the thermal denaturation in the presence of the extrinsic fluorescent dye SYPRO Orange (Sigma Aldrich, St Louis, MO, USA) using a LightCycler 480 Real-Time PCR System from Roche Applied Science (F. Hoffmann–La Roche Ltd., Basel, Switzerland). 14-3-3γ was prepared at a concentration of 7 µM subunit, customarily in 20 mM Na-Hepes, pH 7.4. 5× SYPRO Orange was added to the samples, and titrations either with the peptides TH-(1-43) and THp-(1-43) or sodium salts of kosmotropic ions (citrate^3−^, SO_4_^2−^, HPO_4_^2−^ and Cl^−^) was performed by adding increasing amounts of stock solutions of either 0.21 mM peptide or 1 M kosmotropic salt, pH 7.4, in a total sample volume of 50 µl. Thermal denaturation was monitored by following the increase in SYPRO Orange fluorescence associated with the protein unfolding (λ_ex_ = 465 nm, λ_em_ = 610 nm) from 20 to 95°C at a scan rate of 2°C/min with data pitch of 0.2°C.

The effect of the ligand on the midpoint melting temperature (*T*_m_) was analyzed as described by Cooper and McAuley-Hecht [31], using the equation
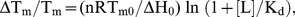
(1)where Δ*H*_0_ is the enthalpy of the transition, *T*_m0_ is the midpoint temperature in the absence of ligand, Δ*T*_m_ ( = *T*_m_−*T*_m0_) is the shift in transition temperature of the protein brought about by the presence of ligand (L), *K*_d_ is the intrinsic dissociation constant, *n* is the number of ligand binding sites and *R* is the gas constant.

### 31Phosphate-NMR

Spectra of non-dialyzed 14-3-3γ prepared in 10 mM citric acid/Na-citrate buffer, pH 7.3 were acquired at 25°C on a 500 MHz DRX Bruker instrument using a receiver gain of 5 160.6 and accumulating 32 000 transients. The sample had a 14-3-3γ dimer concentration of 0.7 mM, and 5% D_2_O was included in the solvent. The data was exponentially multiplied using a line broadening of 30 Hz prior to Fourier transformation. A reference sample containing only the citric acid/Na-citrate buffer was measured at identical conditions to detect and control for buffer phosphate contaminants.

### Molecular dynamics (MD) simulations

All-atom MD simulations of 14-3-3γ were performed both with and without bound phosphopeptide RAIpSLP, designated holo and apo 14-3-3γ, respectively. The atomic dimeric models were prepared from the high-resolution crystal structures of 14-3-3γ (PDB 2B05, chains B+C) [4]. All atomic models were prepared with Amber 10 [32] and the corresponding Amber99SB forcefield [33], [34]. Protonation states of side chains in the protein and protein-peptide complex were assigned based on the 3D-structure using the well established software PROPKA (see [35] for details) and PDB2PQR at pH 7.0 [36]. Accordingly, the His residues obtained the following protonation states: His158 at N-delta, His164 at N-delta, His195 at N-delta and His169 at N-epsilon. For each of the simulations, the system was implicitly neutralized using *neutralizing plasma*, implemented in Amber10, and the protein was solvated in a periodic truncated octahedron box with TIP3 water molecules [37], providing 16 Å of water between the protein surface and the periodic box edge. The solute was minimized for 10 000 steps, followed by 10 000 steps of minimization of the whole system. The protein was then heated to 100 K with weak restraints for 100 ps, and to 300 K for 200 ps. Equilibration with constant pressure and temperature (NPT) of the system was performed for 2 ns prior to the production run in order to ensure correct density. The production runs lasted for 100 ns and were performed with constant volume and energy (NVE) with a 1 fs time step, using SHAKE constraints on hydrogen-heavy atom bonds. MD simulations of 14-3-3ζ were prepared from PDB 2O02 (chains A+B) [38] with and without bound phosphopeptide (RAIpSLP), designated 14-3-3ζ holo and apo, respectively. Simulations were carried out using the same force field and protocol as described for 14-3-3γ for a production phase of 80 ns. An atomic model of the 14-3-3γ H158F/H195S variant was prepared by manually mutating the corresponding residues in 14-3-3γ. The mutated structure was subsequently minimized for 2 000 steps while holding the non-mutated residues fixed. Following the same equilibration protocol as described for wt-14-3-3γ, the mutated structure was issued to an extra 500 ps simulation with weak restraints on non-mutated residues. An 80 ns long MD simulation of the double mutant H158F/H195S (with bound phosphopeptide (RAIpSLP)) was carried out using the same force field and protocol as described for 14-3-3γ.

### Calculations of electrostatic surface potential

Calculation of the electrostatic potential was carried out with the Adaptive Poisson-Boltzmann Solver (APBS) software [39]. The potential on the solvent accessible surface was visualized in PyMOL, v. 1.4 PyMOL (Schrödinger, LLC, New York, NY, USA). Structures for electrostatic potential calculation were collected after minimization, 50 ns and 80 ns from the respective MD simulations, and also after 100 ns for 14-3-3γ. All collected structures from the MD simulations were issued to 500 steps of minimization, and the bound phosphopeptide was removed prior to the calculation of the electrostatic potential. The protonation states were kept to those assigned to the X-ray structures (see above), except when assessing the pH dependency of the electrostatic potential. For the latter, protonation states were assigned according to p*K*_a_ estimates using PROPKA and PDB2PQR at pH 5.5, 6.0, 6.5, and 7.0. Charges and radii were assigned using the Amber99SB force field [33], [34]. Electrostatic surface potentials were calculated by solving the linearized Poisson-Boltzmann equation at 310 K employing a dielectric constant of 2.0 and 78.0 for the solute and solvent, respectively.

## Results

### The binding of 14-3-3γ to negatively charged membranes; effect of ligand and kosmotropic salts

**Figure 1:**
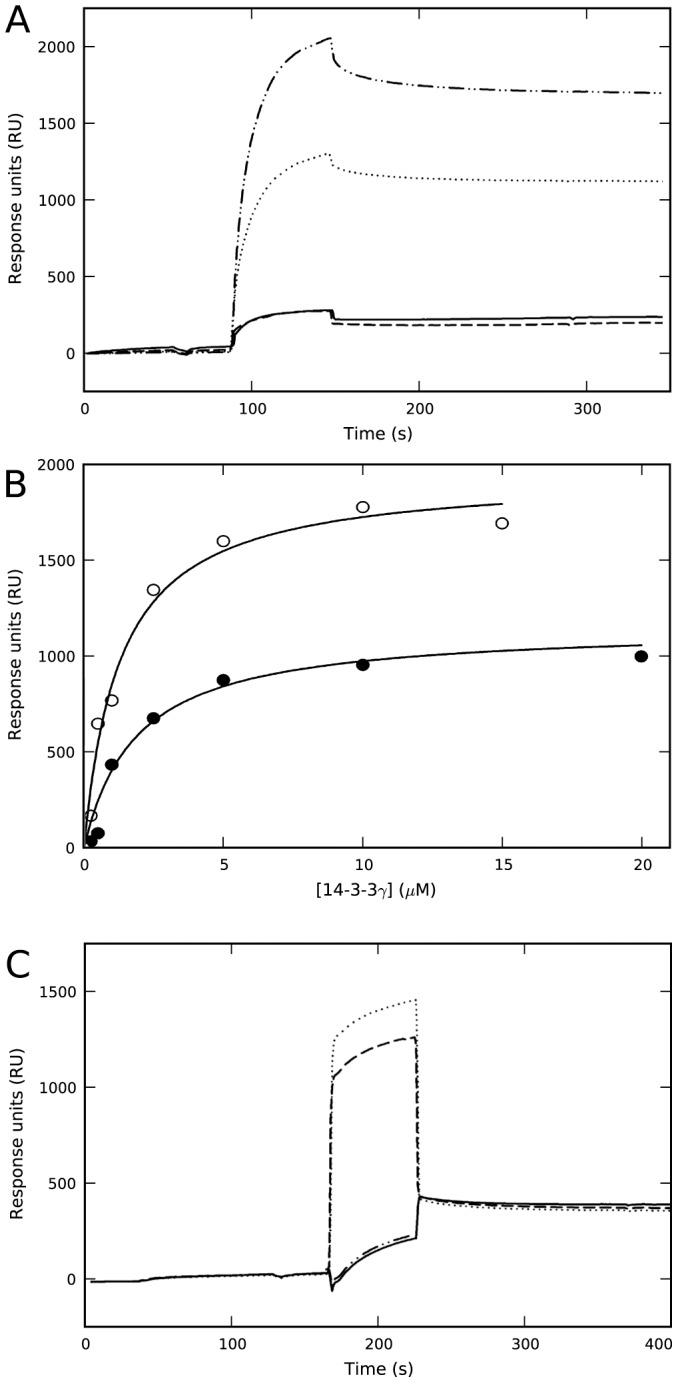
Effect of phosphopeptide ligand and kosmotropic salts on the binding of 14-3-3γ to negatively charged membranes. A) Representative sensorgrams for the binding of 14-3-3γ as purified (10 µM subunit) prepared in HBS-N buffer (10 mM Na-Hepes, pH 7.4, 150 mM NaCl) to liposomes made of PC∶PBPS (⋅⋅⋅⋅⋅⋅⋅⋅), or PC (– – –), and for the binding of 14-3-3γ (10 µM subunit) with THp-(1-43) (20 µM) in HBS-N buffer to liposomes made of PC∶PBPS (–⋅⋅–⋅⋅–) or PC (^_______^). B) The dependence of SPR responses (difference of RUs for binding to liposomes of PC∶PBPS and PC) on the subunit concentration of 14-3-3γ, alone (•) or with 2-fold concentration of THp-(1-43) (○). The binding isotherms were fitted using a single-rectangle, two-parameter equation providing S_0.5_ values of 2.0±0.2 µM for the protein alone and 1.2±0.2 µM for the protein-phosphopeptide complex. C) Sensorgram for the binding to liposomes of PC∶PBPS of d14-3-3γ (extensively dialyzed in 10 mM Na-Hepes, pH 7.4) and further diluted in HBS-N buffer, alone (^_______^) or with a 2-fold concentration of THp-(1-43) (–⋅⋅–⋅⋅–) (running buffer HBS-N in both cases), and with 50 mM Na-phosphate, pH 7.4 (running buffer HBS-N with 50 mM Na-phosphate) (⋅⋅⋅⋅⋅⋅⋅⋅) or 50 mM Na-sulphate, pH 7.4 (running buffer HBS-N with 50 mM Na-sulphate) (– – –).

The membrane-binding ability of 14-3-3γ was studied by SPR using L1 sensor chips coated with liposomes made of both zwitterionic PC and negatively charged PC∶PBPS (1∶1) mixtures. As previously shown [21], [24], purified recombinant 14-3-3γ effectively bound to liposomes made of PC∶PBPS, a composition which seems to properly mimic the charge and fatty acyl composition of synaptic membranes [21], but did not bind to PC-liposomes (Figure 1A). Upon incubation of 14-3-3γ with a 2-fold concentration of phosphorylated THp-(1-43) peptide corresponding to the 14-3-3-interacting domain of TH – conditions at which most THp-(1-43) is bound to 14-3-3γ, with no binding of the peptide itself to the membrane [21] – a higher binding extent is obtained at equal concentration of 14-3-3γ (Figure 1A). Moreover, the affinity of the complex is higher than that of the protein alone (Figure 1B).

In view of the stimulating effect of the ligand on the membrane-binding, we performed an exhaustive dialysis of 14-3-3γ in 10 mM Na-Hepes, pH 7.4, resulting in dialyzed 14-3-3γ (i.e. d14-3-3γ). When membrane-binding was analyzed in the same buffer added 150 mM NaCl, we could not detect any binding of d14-3-3γ to PC∶PBPS-liposomes (Figure 1C). In order to elucidate why binding is abolished upon dialysis, we first investigated the effect of phosphate, and observed that membrane-binding is in fact recovered upon incubation of d14-3-3γ with 50 mM Na-phosphate, pH 7.4 (Figure 1C).

The last steps of purification of 14-3-3γ are performed in 50 mM Na-phosphate, pH 7.4, 150 mM NaCl, a buffer in which the protein was stable, with no tendency to aggregate. Phosphate-NMR investigation of the non-dialyzed protein strongly supported the presence of protein-bound inorganic phosphate in the purified protein sample even after exhaustive up-concentration and dilution in the NMR buffer (Figure S2). The presence of phosphate in 14-3-3γ samples as isolated might explain their membrane-binding ability (Figure 1A) [21]. It has previously been shown that sulphate ions bind at the phosphoserine binding pocket of 14-3-3 at crystallization conditions [40] which might indicate that the phosphate-induced binding of 14-3-3γ to the membranes could be the result of a specific binding of phosphate uniquely at the phosphopeptide binding site. This explanation does not, however, seem plausible since d14-3-3γ, in the absence of salt, did not bind to liposomes regardless of the presence of THp-(1-43) and despite the fact that d14-3-3 proteins bind THp-(1-43) with high affinity at 0 mM salt (K_d_∼0.22 µM by SPR (data not shown) and *K*_d_∼0.19 µM by DSF (see below)). On the other hand, it is well established that phosphate and other kosmotropic salts stabilize proteins as well as protein-membrane interactions, mainly by enhancing hydrophobic interactions and/or screening charged residues [41], [42], [43]. In fact, at pH 7.4 both Na-sulfate (at 50 mM) (Figure 1C) and Na-chloride (at >200 mM) (data not shown) also triggered the binding of d14-3-3γ to PC∶PBPS-liposomes.

### Stabilization of d14-3-3γ by phosphopeptide and kosmotropic salts, as seen by CD and fluorescence

**Figure 2:**
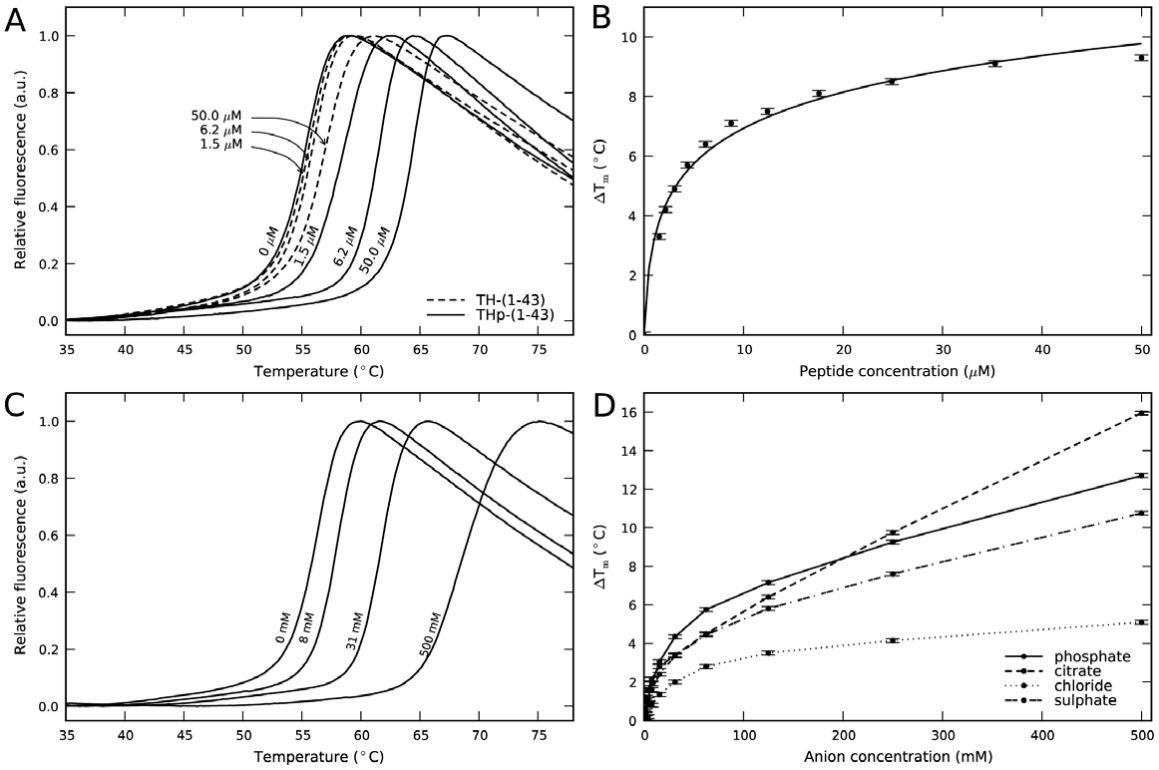
The binding of phosphopeptide ligand and kosmotropic salts to 14-3-3γ by differential scanning fluorimetry (DSF). A) DSF-monitored thermal denaturation of d14-3-3γ, with increasing concentration of Ser19-phosphorylated THp-(1-43) (^_______^) and TH-(1-43) (– – –). B) Concentration dependency of the *T*_m_ changes calculated by DSF for THp-(1-43). Fitting of the data to the equation 1 (see Materials and Methods) [31] (continuous line) provided a *K*_d_∼0.19±0.04 µM. C) DSF-monitored thermal denaturation of d14-3-3γ, with increasing concentrations of Na-phosphate. D) Concentration dependency of the *T*_m_ changes calculated by DSF for Na-citrate (– – –), Na-sulphate (–⋅–⋅–), Na-phosphate (^_______^) and Na-chloride (⋅⋅⋅⋅⋅⋅⋅⋅). For all panels, d14-3-3γ (10 µM) subunit was prepared in 10 mM Na-Hepes, pH 7.4, 150 mM NaCl, and increasing concentrations of peptides from a solution of 0.21 mM (A, B) or kosmotropic salts from a solution 1 M, pH 7.4 (C, D) were added.

Putative conformational effects of the phosphopeptide THp-(1-43) on 14-3-3γ were then investigated. The CD spectrum of 14-3-3γ exhibits minima at 208 and 222 nm (Figure S3A), consistent with the crystal structure of all 14-3-3 proteins, where each subunit is formed by nine antiparallel α-helices (Figure S1). Processing the spectra with the CDNN software [44] provided a 68±2% α-helix content, which agrees well with the high helical content (77%) calculated by DSSP [45] on the crystal structure (PDB 2B05). The extensively dialyzed d14-3-3γ displays a similar spectrum (Figure S3A), which is not affected by addition of the peptides TH-(1-43) and THp-(1-43), up to 30 µM, or of Na-phosphate, up to 160 mM. However, both THp-(1-43) and Na-phosphate largely enhanced the thermal stability of d14-3-3γ (Figure S3B and data not shown). A more detailed analysis of the concentration dependency was performed by DSF, which allows measurements with higher concentrations of peptides and salts. Titration with TH-(1-43) had a minor effect on the *T*_m_ of d14-3-3γ, while binding of THp-(1-43) certainly increased the *T*_m_ in a concentration-dependent manner. Fitting of the data to equation 1 provided a *K*_d_∼0.19±0.04 µM (Figure 2A, B). Likewise, d14-3-3γ was stabilized by Na-phosphate and other sodium salts of kosmotropic ions, but at higher concentration ranges than THp-(1-43) (Figure 2C, D), similar to those required for the protein-membrane interactions (Figure 1C). The concentration dependency of the *T*_m_-increase for each salt, i.e. Na-citrate>Na-phosphate≈Na-sulphate>Na-chloride, resembles the Hofmeister series order [41], [46].

### Structural and electrostatic analysis; molecular dynamics (MD) simulations

**Figure 3:**
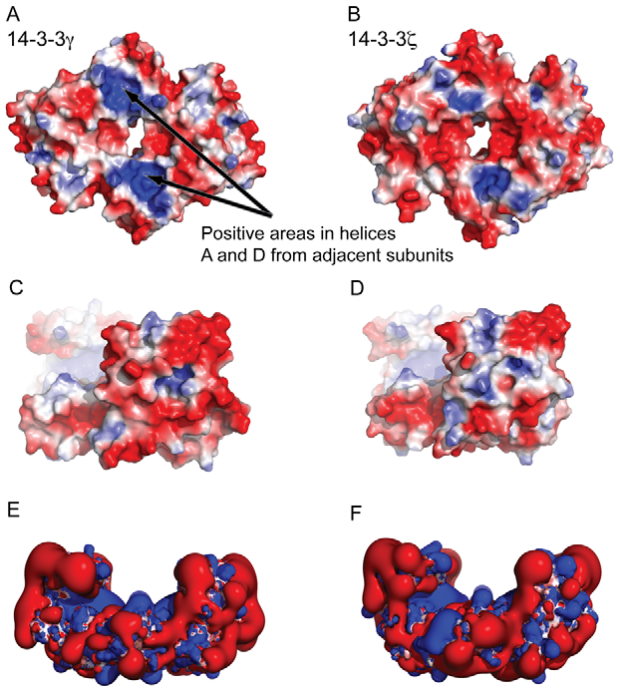
Representation of electrostatic surface potentials. The electrostatic potentials are calculated on the X-ray structures of 14-3-3γ (PDB 2B05) (A, C, E) and 14-3-3ζ (PDB 2O02) (B, D, F). The electrostatic potentials are visualized on the solvent accessible surface in panel A–D with values colored from blue to red (from +2 kT/e to −2 kT/e). Panels E and F show the iso-contours of the electrostatic potential (+1 kT/e to −1 kT/e). The area with stronger positive electrostatic potential in 14-3-3γ (A) compared with 14-3-3ζ (B) corresponds to residues in helices A, B and D in the dimerization region that involves the N-terminal from each subunit (Figure S1). Positive areas are predicted to contact the membrane in an initial phase driven by the electrostatics, while the negative areas on the convex side of the protein are possibly involved in tuning the orientation of the positive dimerization region towards the membrane. The bound phosphopeptides were omitted prior to the calculations of the electrostatic potentials.

To get insights into the structural basis for the interaction of 14-3-3γ with membranes, we first analyzed the structural differences between 14-3-3γ and another 14-3-3 isoform that does not bind to membranes at neutral pH, i.e. 14-3-3ζ [19], [20], [21], [24]. Analysis of the electrostatic surface potential of the crystal structures of these two isoforms (PDB 2B05 for holo-14-3-3γ and PDB 2O02 for holo-14-3-3ζ), at pH 7.0, revealed features that might be related to the specific membrane-binding capability of 14-3-3γ. We observed a stronger positive electrostatic potential in helices A and B and part of helix D in the structure of the γ-isoform (Figure 3A) than in the ζ-isoform (Figure 3B). These helices form the N-terminal dimerization region from each subunit [47] (Figure S1), and might be important to interact with the negatively charged membrane in an initial electrostatic phase. The area surrounding the dimerization region, and towards the laterals of the convex side, presents a negative surface potential in both isoforms, in agreement with the acidic character of all the members of the 14-3-3 protein family (Figure 3C–F).

**Figure 4:**
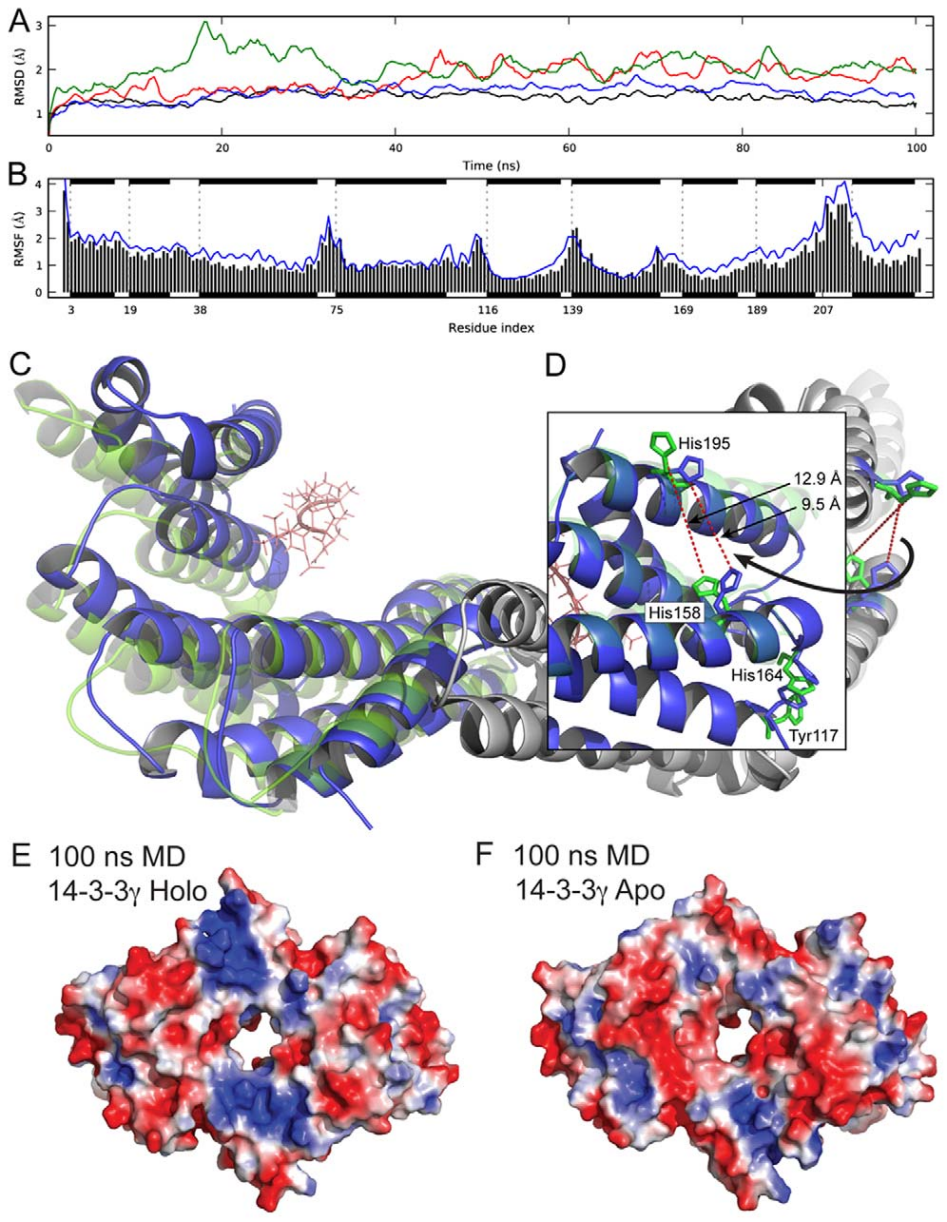
MD simulations of phosphopeptide-bound (holo-14-3-3γ) and ligand-free (apo-14-3-3) structures. A) Structural deviation from the initial X-ray structure (PDB 2B05) along the simulation time (100 ns) for phosphopeptide (RAIpSLP)-free apo (green and red lines, one line for each monomer A and B) and phosphopeptide-bound holo (blue and black lines for each monomer); RMSD, root mean square deviation. B) Positional fluctuations (root mean square fluctuations (RMSF)) along the holo (black bars) and apo (blue lines) 14-3-3γ. Helices are indicated schematically as black stripes. C) Backbone ribbon overlay representation of the dimeric structures obtained at the end of the MD simulations (100 ns) of holo (blue) and apo (green) 14-3-3γ. The inset (D) shows the residues mutated in this study (Tyr117, His158, His164 and His195) in stick representation, and the averaged distance between His158 and His195 at the end of the simulations. E, F) Representation of surface electrostatic potentials, calculated on the solvent accessible surface of the MD simulated structures of holo (E) and apo (F) 14-3-3γ, colored from blue to red (from +2 kT/e to −2 kT/e).

**Figure 5:**
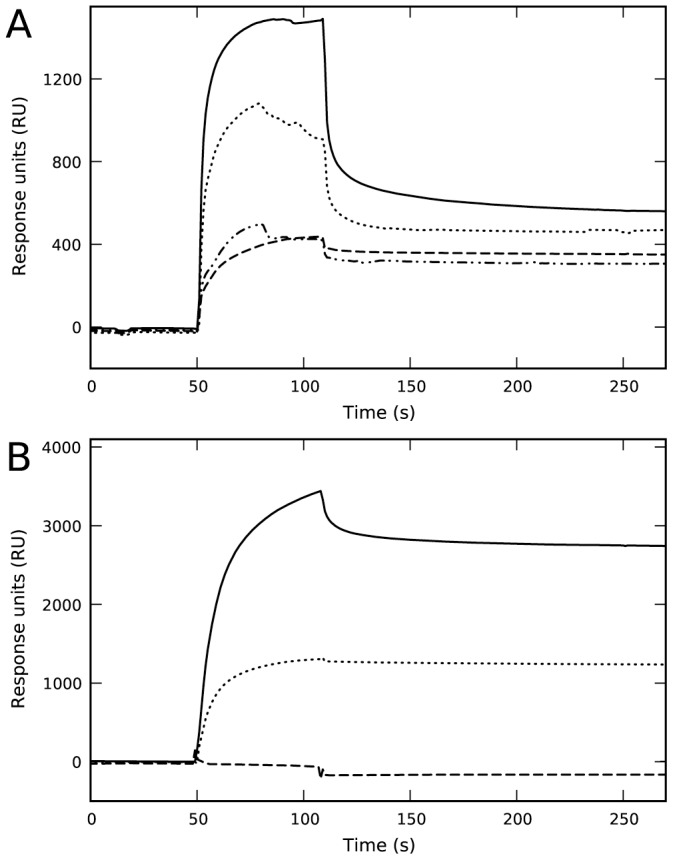
Effect of mutation of specific histidine residues and of pH on the binding of 14-3-3γ to liposomes of PC∶PBPS (1∶1) measured by surface plasmon resonance (SPR). A) Representative sensorgrams for the binding of H158F- (⋅⋅⋅⋅⋅⋅⋅⋅), H195S- (–⋅⋅–⋅⋅–) and H158F/H195S- (– – –), comparative to wt-14-3-3γ (^_______^). The sensorgrams for H164E- and Y117F-14-3-3γ were similar to that of wt-14-3-3γ. The protein samples were prepared in 100 mM Na-phosphate, pH 7.4. B) Representative sensorgrams for the binding of wt-14-3-3γ as isolated, and diluted in either 100 mM Na-phosphate, pH 6.0 (^_______^), 100 mM Na-phosphate, pH 7.0 (⋅⋅⋅⋅⋅⋅⋅⋅), or 100 mM Tris-HCl, pH 8.0 (– – –). For both (A) and (B), liposomes were immobilized on the sensor chip at 4000–6000 RU, and each protein was applied at ∼50 µM subunit and injected at 25°C. The sample preparation buffers were used as running buffer.

With respect to the enhancement of membrane binding induced by the phosphopeptide (Figure 1B), it is not straightforward to elucidate the mechanistic details of this stimulation, since only the phosphopeptide-bound holo-structure of 14-3-3γ has been solved by X-ray crystallography [4], [47] (Figure S1). However, both apo- and holo-structures have been obtained for other isoforms [2], [4], [47], providing templates to interpret possible ligand-induced conformational changes. We thus probed the conformational dynamics and the effects of ligand binding by performing MD simulations for 14-3-3γ and 14-3-3ζ, both when bound to the phosphopeptides and when the phosphopeptide was deleted from the initial structure (then referred to as apo-14-3-3γ or apo-14-3-3ζ). All systems were stable along the 80 ns-long MD simulations, which was extended to 100 ns for 14-3-3γ (Figure 4A). However, apo-14-3-3γ shows larger structural fluctuation mainly in the helix-connecting loops and the C-terminal helices (Figures 4A, B and Figure S4; see also PDB File S1 and PDB File S2, for MD-simulated (100 ns) holo- and apo-14-3-3γ, respectively), in correlation with the lower stability of the apo- compared with the holo-form, as observed by DSF (Figure 2A). Furthermore, the apo-simulation exhibits an increased deviation from the initial structure (Figure 4A), adopting a more open conformation with respect to the central cavity (Figure 4C). This resembles the opening observed in the crystal structures of apo-14-3-3β (PDB 2BQ0; subunit B) when compared with the holo-form (PDB 2C23) [4]. Accordingly, the RMSD value for simulated apo-14-3-3γ *vs.* apo-14-3-3β decreases from 2.8 Å to 1.5 Å along the MD simulation, while the holo-14-3-3γ simulation samples the conformational space close to the initial holo X-ray structure (Figure 4A). Moreover, the MD simulated ligand-bound holo-14-3-3γ shows slightly larger protrusion of the helices A, B and D towards the convex side (downwards in Figure 4C) than the apo-structure. Many of these conformational features are also observed in the corresponding holo- and apo-simulations of 14-3-3ζ (80 ns long). Interestingly, the MD simulated apo-14-3-3γ shows a weaker positive electrostatic potential in the dimerization region than both the crystal structure of holo-14-3-3γ (Figure 3A) and the MD simulated holo-structure (Figures 4E, F), while the MD-simulated structures of both apo- and holo-14-3-3ζ (80 ns long) reveal a more stable potential throughout the simulation (Figure S5). Moreover, the two positive patches in the dimerization domain gradually become fainter along the apo-simulation of 14-3-3γ (Figure 5S).

The observed differences in surface electrostatic potential for the crystal structures of 14-3-3γ- and ζ-isoforms, as well as along the MD simulations of the apo- and holo-forms of 14-3-3γ, might be related to a γ-specific spatial redistribution of charges upon ligand binding. There are many solvent-exposed acidic residues on the convex surface of 14-3-3γ which might have a role to discriminate and tune the positively charged dimerization area in the initial stages of binding. Moreover, most of the acidic residues, as well as most of the basic residues, are conserved between the γ and either the ζ or other non-membrane binding 14-3-3 isoforms, e.g. η [21] (Figure S1). Nevertheless, we identified a few non-conserved, 14-3-3γ-specific ionizable residues, i.e. three non-conserved histidine residues His158, His164 and His195 (Figure S1), in addition to Lys152. These residues are all located in the convex side and outside the N-terminal dimerization region. His158 and His195, but not His164 or Lys152, experienced conformational changes along the simulation toward the apo-form, since they are located in two helices that approach each other in the ligand-bound state (Figure 4C, D).

### Site-directed mutagenesis of residues of 14-3-3γ predicted to be involved in membrane association

In order to experimentally probe if non-conserved ionizable residues are crucial for membrane binding, we performed site-directed mutagenesis and investigated the membrane binding ability of the mutants. Histidine residues have been associated with pH-dependent modulations of protein-protein [48], [49] and protein-membrane interactions [50], [51]. Initially, we restricted our choice to histidines, since their imidazole side chain is prone to change of charge at physiologically relevant pH-values upon perturbations in their local environments. The predicted p*K*_a_ values in the holo-14-3-3γ structure were 4.8 for His158, and 6.8 and 6.4 for His164 and His195, respectively, indicating that the two latter residues can get protonated following small pH shifts towards the acidic range. We mutated these three histidine residues to the corresponding substitutions in human 14-3-3ζ (Figure S1), and prepared H158F-, H164E- and H195S-14-3-3γ. We also prepared the mutant Y117F, since the non-conserved Tyr117 π-stacks with His164 (Figure 4D) and might modulate the p*K*_a_-value of the histidine, as described in other systems [52]. The four mutants were purified at similar yields as wt-14-3-3γ and also showed similar affinity for binding the phosphopeptide THp-(1-43) as wt (Figure 2A and data not shown). Moreover, H164E and Y117F showed a similar binding response to PC∶PBPS liposomes as wt-14-3-3γ in the presence of phosphate. On the other hand, the membrane binding ability of the H158F and H195S mutants, notably the latter, experienced a large decrease (Figure 5A). Finally, we prepared the double mutant H158F/H195S-14-3-3γ, which was essentially devoid of membrane-binding ability (Figure 5A).

**Figure 6:**
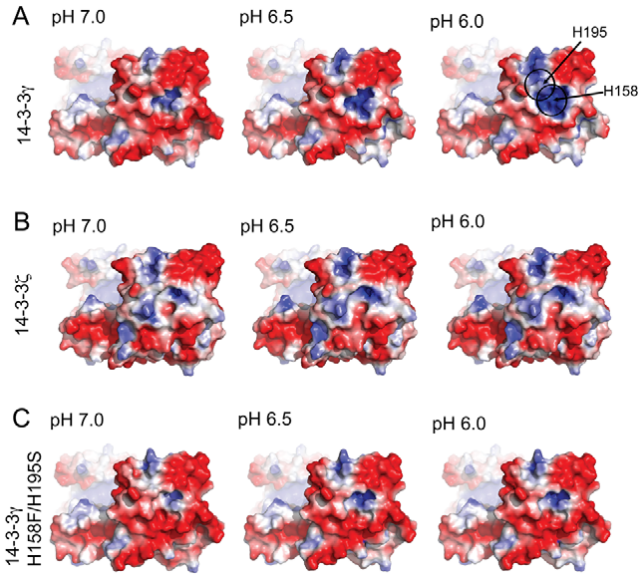
pH dependency of the electrostatic potential at the convex lateral side. The electrostatic potential is shown at pH values 7.0, 6.5, and 6.0 (column wise), for 14-3-3γ (A), 14-3-3ζ (B), and H158F/H195S-14-3-3γ (C).

In order to further investigate in which way His195 and His158 might contribute to the membrane binding of the γ-isoform we first prepared the structural model of the H158F/H195S-14-3-3γ double mutant in the phosphopeptide-bound (holo) state, and run an 80 ns MD simulation. The structural dynamics of this protein variant along MD did, as expected, not deviate significantly from the holo-14-3-3γ simulation. Moreover, at the end of the simulation, and at pH 7.0, the surface electrostatic potential around the dimerization domain was very similar between the wt and the mutant (Figure S5). We then performed a comparative pH dependent electrostatic analysis between the final MD simulated holo-structures of 14-3-3γ, 14-3-3ζ and H158F/H195S-14-3-3γ in order to probe if an increased protonation of His158 and His195 might play a role in the membrane-binding process. Previous studies have shown that the protein would orient tangential to the membrane through the convex side, without significant penetration in the bilayer [24]. Thus, according to the pH gradient at the surface of a negatively charged membrane [53], the convex side of the protein is expected to experience a decrease in local pH from the bulk pH 7.0 to around 5.5–6.0. We therefore analyzed the pH dependent surface electrostatic potential at pH 7.0, 6.5, 6.0 and 5.5, in the simulated structures (Figure 6; see also Figure S6 for more detailed analyses). Upon pH reduction an increasing cationic patch appears in wt-14-3-3γ around His158 and His195, but not in 14-3-3ζ or H158F/H195S-14-3-3γ (Figure 6). At pH 6.0, the protonation is complete for His195 (Figure S6), and at this pH a large increase in membrane binding was also measured by SPR for wt-14-3-γ (Figure 5B).

## Discussion

### 14-3-3γ as a peripheral protein

Peripheral proteins interact with membranes using a number of strategies, e.g. specific lipid-targeting domains [28] or covalent lipid anchors (e.g. myristoyl or prenyl) that embed in the membrane [54], [55]. There is also a large number of proteins that are recruited to the membrane through changes in their conformation and surface electrostatic potential, often involving amphipathic helices in the interacting interfaces [26], [27]. Peripheral proteins reversibly modulate membrane interaction through reorganization of the lipid composition of the membrane and/or – in the case of proteins using lipid anchors – by reverting the posttranslational modification or through conformational changes that hide the lipid tag. In this respect, the effect of ligand binding such as Ca^2+^ or nucleotides (e.g. GTP) on the modulation of membrane binding is well established [56], [57]. In this work we show that the ability of 14-3-3γ to interact with lipid bilayers is enhanced in the presence of the phosphopeptide ligand. Moreover, binding does not involve lipid tags. Instead, membrane binding appears to involve specific motifs, mainly amphipathic helices at the N-terminal dimerization area and two histidine residues at the convex lateral sides.

Our results indicate that the initial electrostatic interaction of 14-3-3γ with the membrane through the N-terminal dimerization domain would be favored by binding of the phosphopeptide ligand, a binding that increases the cationic character of the dimerization area. The involvement of the N-terminal region is in agreement with results obtained with 14-3-3ε by Jones *et al.*, who through truncation mutagenesis, proteolysis and immunodetection also concluded that the 12 kDa-N-terminal region (corresponding to helices A to D) is implicated in membrane interaction [22]. 14-3-3ε is the other isoform in addition to γ, that shows significant binding to membranes [19], [20], [22], and it is interesting that 14-3-3ε has an arginine residue in the position corresponding to His195 in 14-3-3γ (Figure S1).

In a further consolidating stage, the interaction with the membrane might be stabilized through hydrophobic interactions involving residues from the N-terminal amphipathic helices and longer-range electrostatic interactions involving cationic patches around the γ-specific histidine residues, which become protonated at pH 6.0 (see below).

The proposed two-step process for membrane-binding of 14-3-3γ is in agreement with binding mechanisms put forward for peripheral and amphitropic proteins devoid of specific tags or lipidic modifications, where initial electrostatic forces localize the protein to the membrane and, subsequently, specific hydrophobic interactions and additional electrostatic forces consolidate membrane-association [21],[58],[59] (see Figure S7 for a model on the predicted orientation of the protein prior to membrane interaction). The binding of peripheral proteins often involves charged side-chains at amphipathic helices that can establish both initial electrostatic and hydrophobic interactions with membranes [58], [60]. Such helices often modulate membrane physical properties [61], [62], and are optimal structural candidates for reversible binding to membranes [27], [63]. Helices A, B and D (Figure S1) of 14-3-3γ are amphipathic and could mediate the initial electrostatic interaction of the holo-protein and, by a further rotation, also readily intercalate their hydrophobic area in the bilayer, a process that would be favored in the presence of salts.

### The effect of kosmotropic salts on the interaction of 14-3-3γ with the membrane

Kosmotropic salts induce stabilizing conformational changes in proteins [64], [65], [66] and also bind and affect the structure and stability of the lipid bilayer [67]. Direct ion-macromolecule interactions, rather than their effect on the bulk water structure, are largely responsible for these effects [46]. Moreover, kosmotropic salts stabilize protein-membrane complexes and have been found to induce the active conformation in membrane-bound receptors [43], most probably by increasing the interfacial tension between protein and solvent and strengthening the hydrophobic effect [46]. Salts may in fact participate in protein-membrane interactions through screening of (unfavorable) charged residues [41], [43]. Our results show that salts do not induce large conformational changes, but stabilize 14-3-3γ, resembling the effect of the phosphopeptide THp-(1-43) (Figure 2). On the other hand, the binding of the phosphopeptide favors membrane interaction of 14-3-3γ, but it is not sufficient to provide a measurable binding in a salt free solution, while the salts are essential in this respect. The absolute requirement for salt might thus be related to both steps in the proposed membrane binding mode; *initial electrostatic interaction*, requiring the participation of counterpart ions screening the acidic surrounding of the cationic surface of the A, B and D amphipathic helices, and the *consolidation* of the interaction through among other *hydrophobic residues* in the same helices [46]. This sequence of steps is common for protein-membrane interactions involving amphipathic helices [26], [63].

### Histidine residues in electrostatics and membrane interaction of 14-3-3γ

Histidine residues have been associated with pH-dependent modulations of protein-protein [48], [49] and protein-membrane interactions [50], [51]. In such instances histidines function as sensors or switches that protonate as the pH drops (e.g. in the vicinity of a negatively charged membrane [53]), affecting the intermolecular interactions electrostatically. In most cases, the low-pH histidine sensors are either located in the interaction area [50], [68] or modulate this area upon protonation [48]. Residues His158 and His195, proven here by site-directed mutagenesis to be involved in membrane interaction of 14-3-3γ, do not appear to be located in the main interaction area (Figure S7). Rather, His158 and His195 might be involved in an electrostatic switch that operates at neutral-acidic pH following the initial interaction of the protein with the membrane through the cationic dimerization region. Thus, concomitant to the expected decrease in pH in the vicinity of the negatively charge membrane, these histidine residues will increase their protonation, providing additional electrostatic anchors. Unit-charge interactions are long-range (up to a few nm); therefore, the residues do not need to touch the bilayer to contribute to a stable complex. Electrostatic switches may also contribute to the binding by initiating longer range net electrostatic attraction and a reorganization of the protein structure leading to further charge relocation and binding to the membrane [26].

In conclusion, we here show that the interaction of 14-3-3γ with the membrane is favored by binding of phosphorylated-ligand and is dependent on the presence of salts. Amphipathic helices at the cationic N-terminal dimerization region and γ-isoform specific histidine residues appear to be membrane-targeting motifs. Whilst a detailed mechanistic binding model must await the structural elucidation of 14-3-3γ bound to the membrane, our findings contribute to understand the peripheral membrane-binding of the γ-isoform, and suggest a possible role of this regulatory protein in the modulation of the subcellular distribution of its partners. In this context our interest is centered on TH, an enzyme very abundant in dopaminergic neurons and chromaffin cells of the adrenal medulla. TH is essentially soluble and cytoplasmic, but a fraction is also found as membrane-bound in its neuroendocrine locations, mainly at synaptic vesicles and secretory chromaffin granules [69], [70], [71].

## Supporting Information

Figure S1 **The structure of 14-3-3γ and sequence diversity in the 14-3-3 family.** Top) The structure of 14-3-3γ with a phosphoserine (pSer, in red) peptide (RAIpSLP) bound in the concave cavity of each subunit. The dimeric structure of the protein (PDB 2B05) is shown in backbone ribbon (subunit A) and surface representation (subunit B). Down) Sequence alignment (by ClustalW) of all seven human 14-3-3 isoforms, including α-helices A-I. Conserved residues are shown in green and histidine residues that in 14-3-3γ have switched from neutral or negative charged residues (i.e. His158, His164 and His195) are indicated in red.
(TIF)

Figure S2 **^31^Phosphate-NMR.** Spectra of non-dialyzed 14-3-3γ prepared in 10 mM citric acid/Na-citrate buffer, pH 7.3 (red trace). The sample had a 14-3-3 dimer concentration of 0.7 mM, and 5% D_2_O was added to the sample. ^31^P NMR was performed at 25°C on a 500 MHz DRX Bruker instrument using a receiver gain of 5 160.6 and accumulating 32 000 transients. The data was exponentially multiplied using a line broadening of 30 Hz prior to Fourier transformation. A reference sample containing only the citric acid/Na-citrate buffer was measured at identical conditions (black trace). The sample containing 14-3-3γ has a broad ^31^P signal non-attributable to buffer contaminants and that is consistent with a population of phosphates bound to the protein.
(TIF)

Figure S3 **Circular dichroism (CD) of 14-3-3γ and d14-3-3γ; effect of Na-phosphate.** A) Far-UV CD spectrum of 14-3-3γ (7 µM subunit) in 50 mM Na-phosphate, pH 7.4, 150 mM NaCl (black line), d14-3-3γ, after dialysis in 10 mM Na-Hepes, pH 7.4 (blue line) and d14-3-3γ in the presence of 159 mM Na-phosphate (red line). B) CD-monitored thermal denaturation of d14-3-3γ, with increasing concentration of Na-phosphate up to 159 mM. The protein was prepared initially in 10 mM Na-Hepes, pH 7.4, 150 mM NaCl, at 10 µM subunit; increasing concentrations of phosphate were added (from a stock solution of 1 M phosphate, pH 7.4).
(TIF)

Figure S4 **Comparison of positional fluctuations.** The upper panel shows the theoretical positional fluctuations obtained from molecular dynamics simulations along 14-3-3γ-holo (black bars) and 14-3-3γ-apo (red lines); same as main Figure 4B. Helices are indicated schematically as black stripes. The lower panel shows the experimentally obtained beta factors (PDB 2B05).
(TIF)

Figure S5 **Electrostatic potential of 14-3-3 along molecular dynamics (MD) simulations.** The electrostatic potential of representative snapshots obtained from the MD simulations of 14-3-3γ (rows 1–2), 14-3-3ζ (rows 3–4), and H158F/H195S-14-3-3γ (row 5) are visualized on the solvent accessible, convex surface, oriented to visualize the dimerization domain. Values are represented with a color range spanning from red (negative, +2 kT/e) to blue (positive; +2 kT/e) through white (neutral). N/A, not applicable.
(TIF)

Figure S6 **pH dependency of the electrostatic potential at the convex side.** The electrostatic potential is shown at pH values 7.0, 6.5, 6.0 and 5.5 (column wise) for 14-3-3γ (A), 14-3-3ζ (B), and H158F/H195S-14-3-3γ (C).
(TIF)

Figure S7 **Model of the predicted orientation of ligand-bound 14-3-3γ for optimal interaction with negatively charged membranes.** The protein has a positive surface electrostatic potential at the N-terminal dimerization region especially in the presence of bound phosphopeptide (Figure 4), which gives the acidic protein adequate properties for membrane interaction and subsequent intercalation through amphipathic helices A, B and D. The proximity to the membrane also induces the appearance of cationic patches around His158 and His195, aiding to stabilize the membrane bound conformation.
(TIF)

PDB File S1 Dimeric structure obtained at the end of the MD simulation (100 ns) of holo-14-3-3γ. See main text for details.
(PDB)

PDB File S2 Dimeric structure obtained at the end of the MD simulations (100 ns) of apo-14-3-3γ. See main text for details.
(PDB)
