# Supplementary figures and images for: The Peripheral Binding of 14-3-3γ to Membranes Involves Isoform-Specific Histidine Residues
Source: PLoS One. 2012 Nov 26;7(11):e49671. doi: 10.1371/journal.pone.0049671 (PMC3506662; doi:10.1371/journal.pone.0049671)

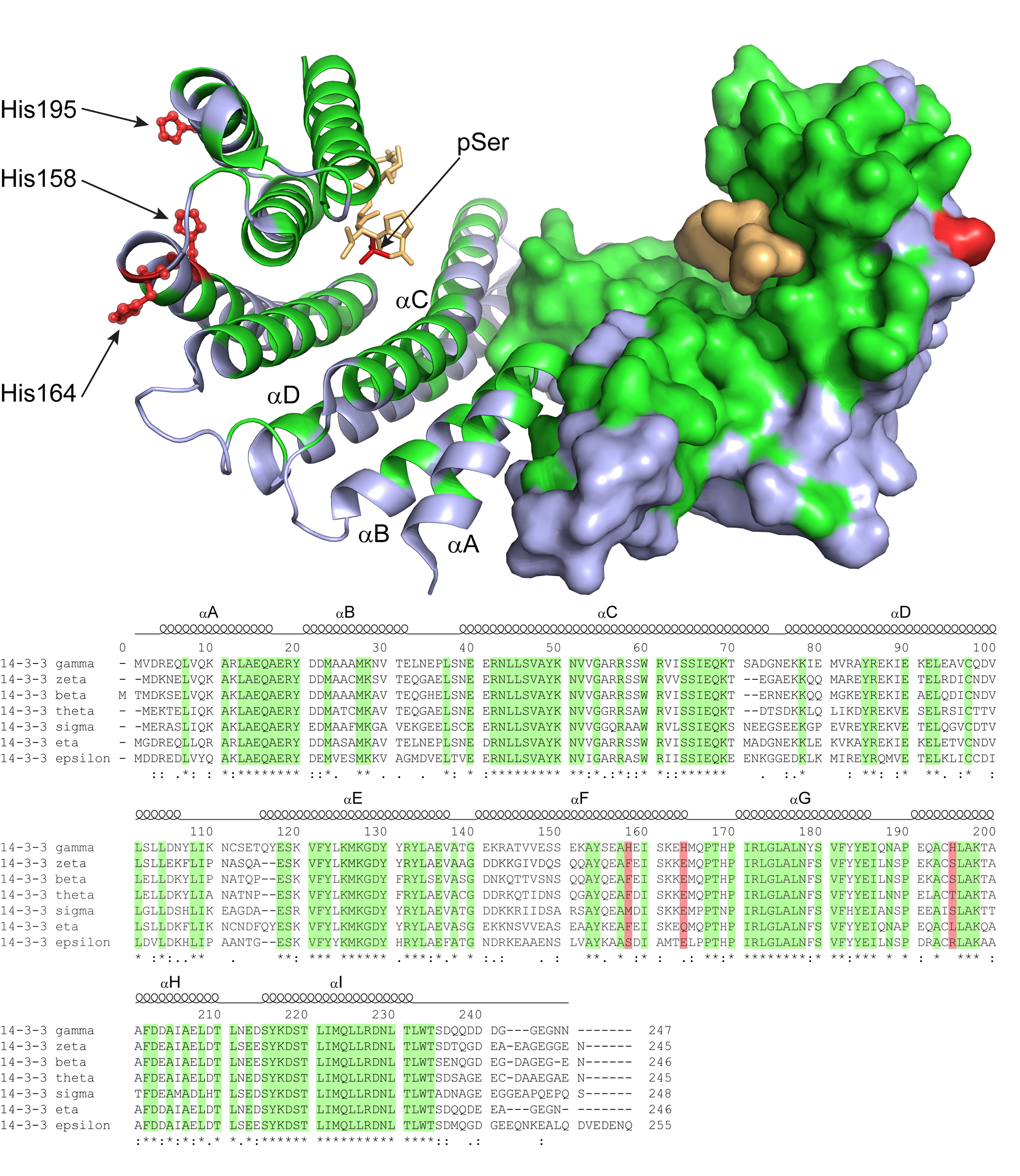

Supplement: Figure S1 — The structure of 14-3-3γ and sequence diversity in the 14-3-3 family. Top) The structure of 14-3-3γ with a phosphoserine (pSer, in red) peptide (RAIpSLP) bound in the concave cavity of each subunit. The dimeric structure of the protein (PDB 2B05) is shown in backbone ribbon (subunit A) and surface representation (subunit B). Down) Sequence alignment (by ClustalW) of all seven human 14-3-3 isoforms, including α-helices A-I. Conserved residues are shown in green and histidine residues that in 14-3-3γ have switched from neutral or negative charged residues (i.e. His158, His164 and His195) are indicated in red. (TIF) [file pone.0049671.s001.tif]

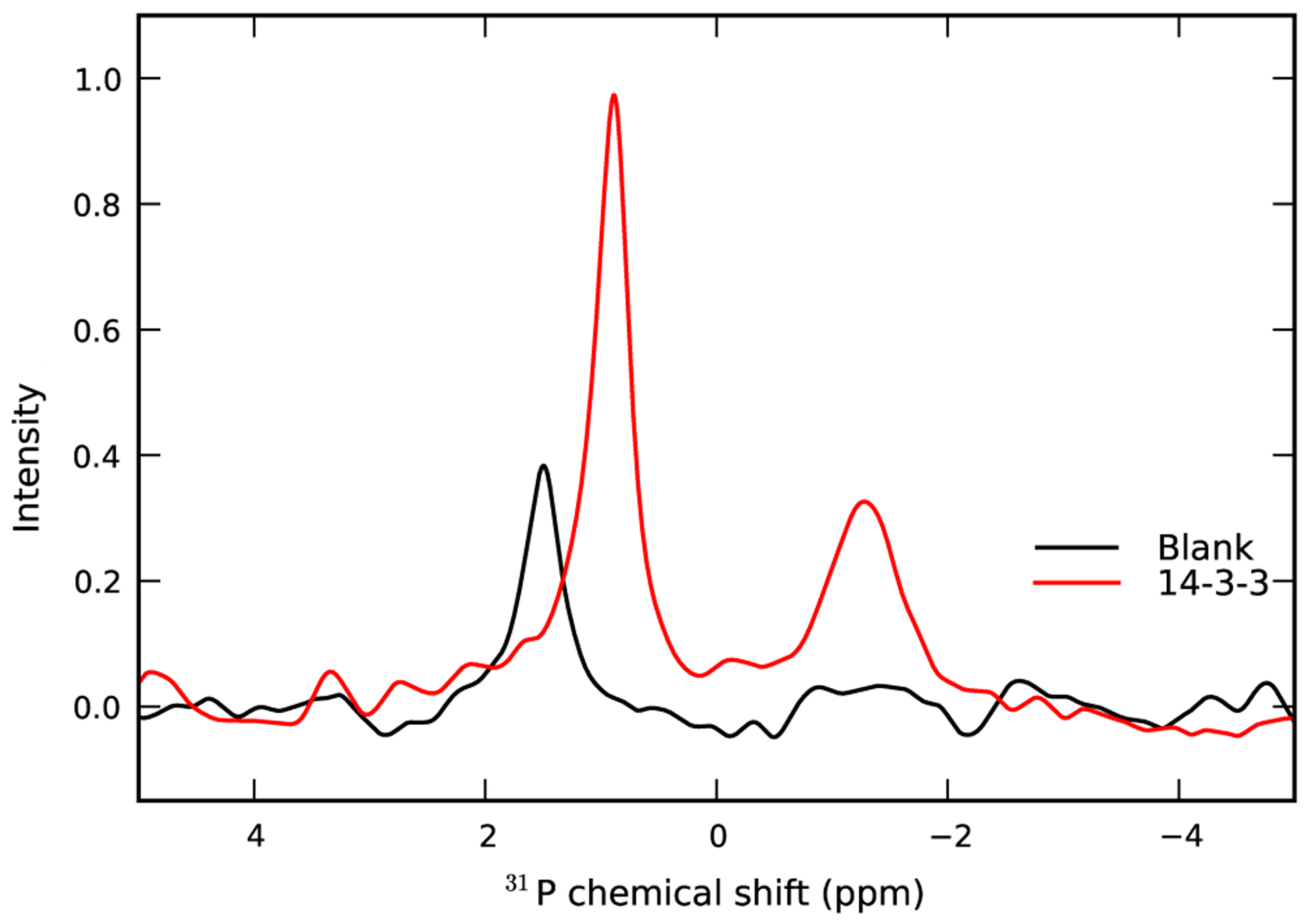

Supplement: Figure S2 — 31Phosphate-NMR. Spectra of non-dialyzed 14-3-3γ prepared in 10 mM citric acid/Na-citrate buffer, pH 7.3 (red trace). The sample had a 14-3-3 dimer concentration of 0.7 mM, and 5% D2O was added to the sample. 31P NMR was performed at 25°C on a 500 MHz DRX Bruker instrument using a receiver gain of 5 160.6 and accumulating 32 000 transients. The data was exponentially multiplied using a line broadening of 30 Hz prior to Fourier transformation. A reference sample containing only the citric acid/Na-citrate buffer was measured at identical conditions (black trace). The sample containing 14-3-3γ has a broad 31P signal non-attributable to buffer contaminants and that is consistent with a population of phosphates bound to the protein. (TIF) [file pone.0049671.s002.tif]

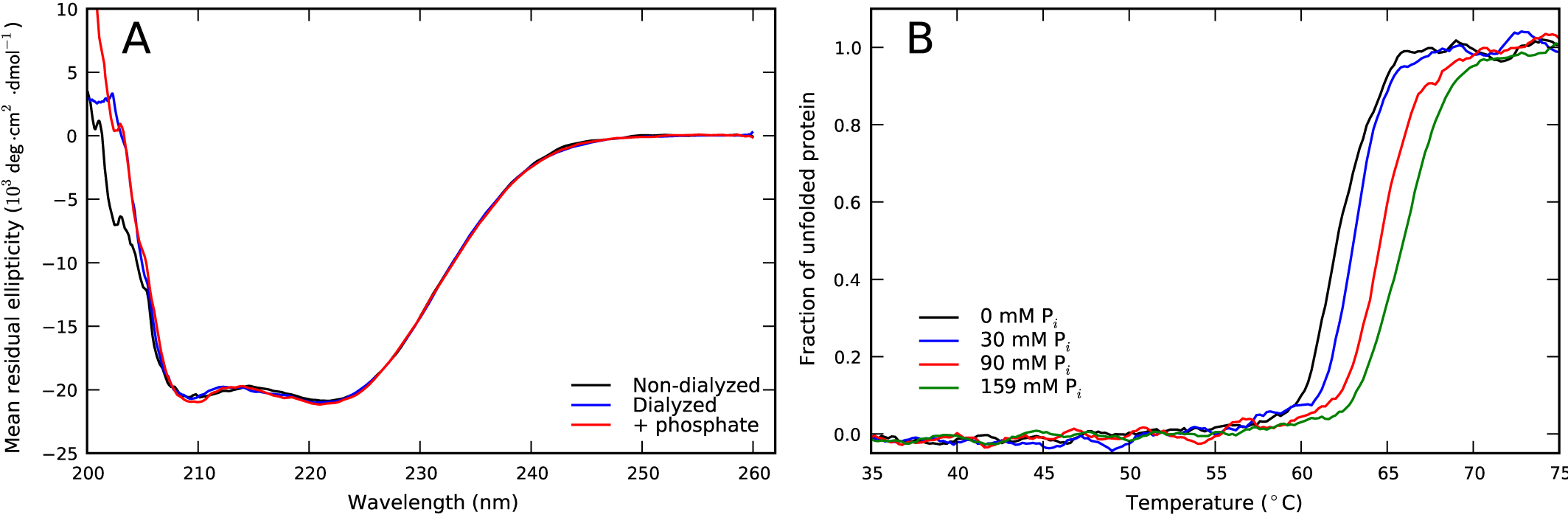

Supplement: Figure S3 — Circular dichroism (CD) of 14-3-3γ and d14-3-3γ; effect of Na-phosphate. A) Far-UV CD spectrum of 14-3-3γ (7 µM subunit) in 50 mM Na-phosphate, pH 7.4, 150 mM NaCl (black line), d14-3-3γ, after dialysis in 10 mM Na-Hepes, pH 7.4 (blue line) and d14-3-3γ in the presence of 159 mM Na-phosphate (red line). B) CD-monitored thermal denaturation of d14-3-3γ, with increasing concentration of Na-phosphate up to 159 mM. The protein was prepared initially in 10 mM Na-Hepes, pH 7.4, 150 mM NaCl, at 10 µM subunit; increasing concentrations of phosphate were added (from a stock solution of 1 M phosphate, pH 7.4). (TIF) [file pone.0049671.s003.tif]

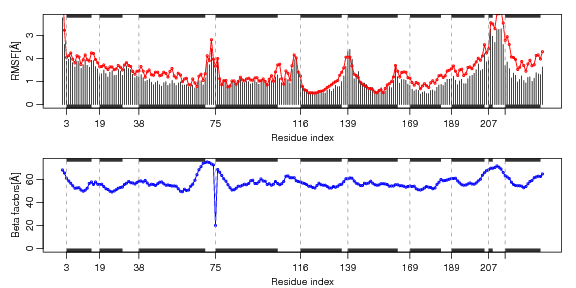

Supplement: Figure S4 — Comparison of positional fluctuations. The upper panel shows the theoretical positional fluctuations obtained from molecular dynamics simulations along 14-3-3γ-holo (black bars) and 14-3-3γ-apo (red lines); same as main Figure 4B. Helices are indicated schematically as black stripes. The lower panel shows the experimentally obtained beta factors (PDB 2B05). (TIF) [file pone.0049671.s004.tif]

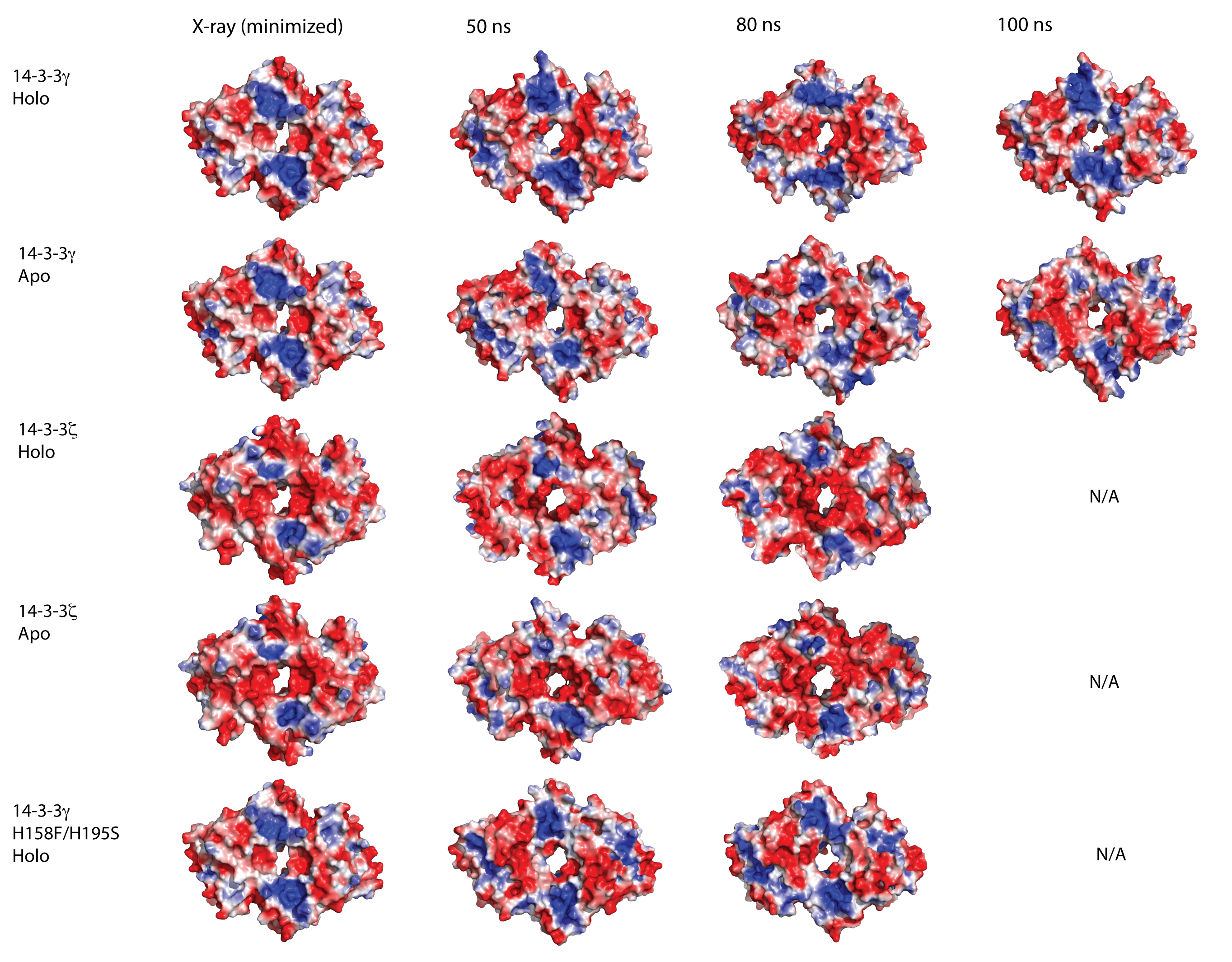

Supplement: Figure S5 — Electrostatic potential of 14-3-3 along molecular dynamics (MD) simulations. The electrostatic potential of representative snapshots obtained from the MD simulations of 14-3-3γ (rows 1–2), 14-3-3ζ (rows 3–4), and H158F/H195S-14-3-3γ (row 5) are visualized on the solvent accessible, convex surface, oriented to visualize the dimerization domain. Values are represented with a color range spanning from red (negative, +2 kT/e) to blue (positive; +2 kT/e) through white (neutral). N/A, not applicable. (TIF) [file pone.0049671.s005.tif]

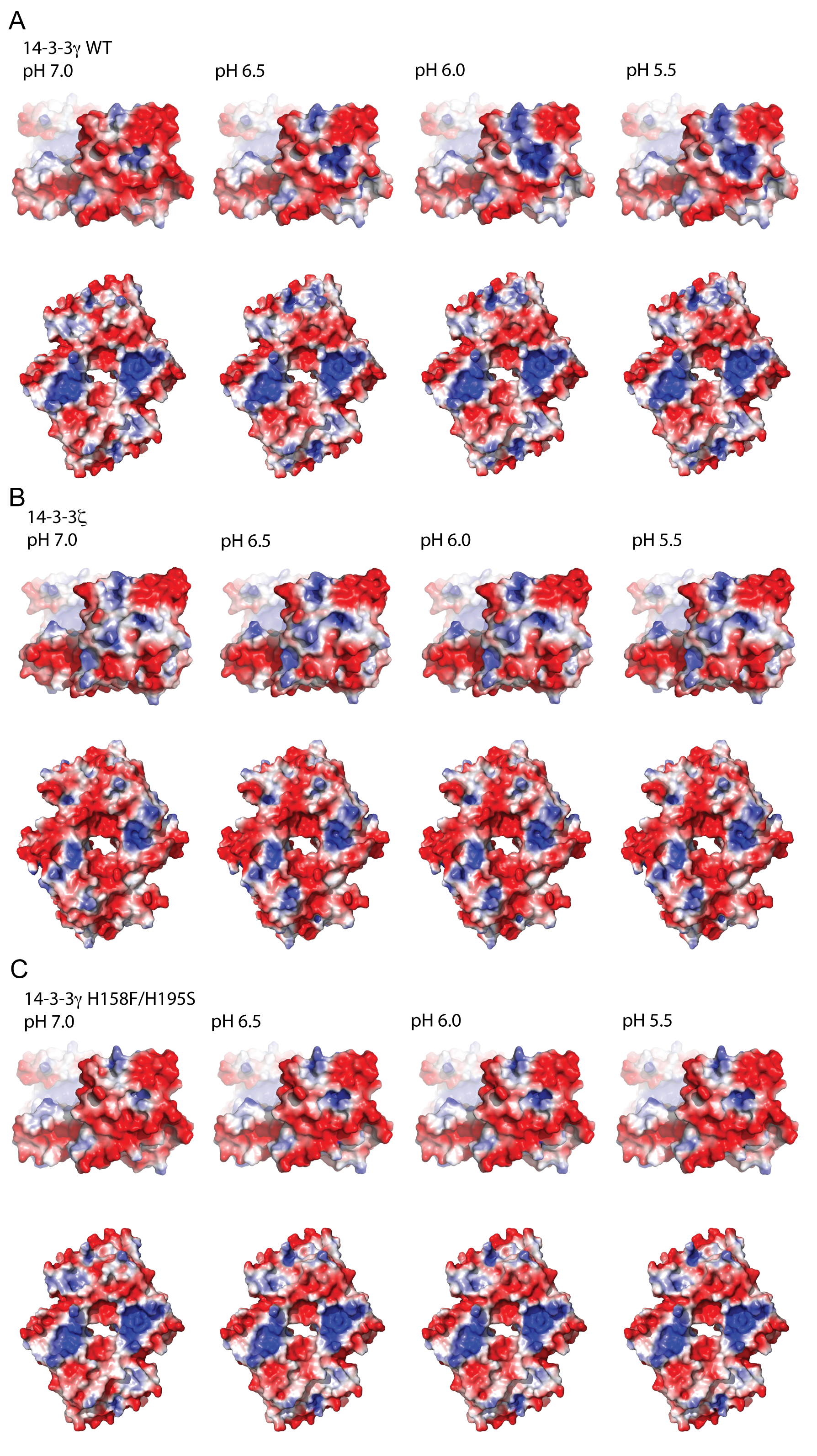

Supplement: Figure S6 — pH dependency of the electrostatic potential at the convex side. The electrostatic potential is shown at pH values 7.0, 6.5, 6.0 and 5.5 (column wise) for 14-3-3γ (A), 14-3-3ζ (B), and H158F/H195S-14-3-3γ (C). (TIF) [file pone.0049671.s006.tif]

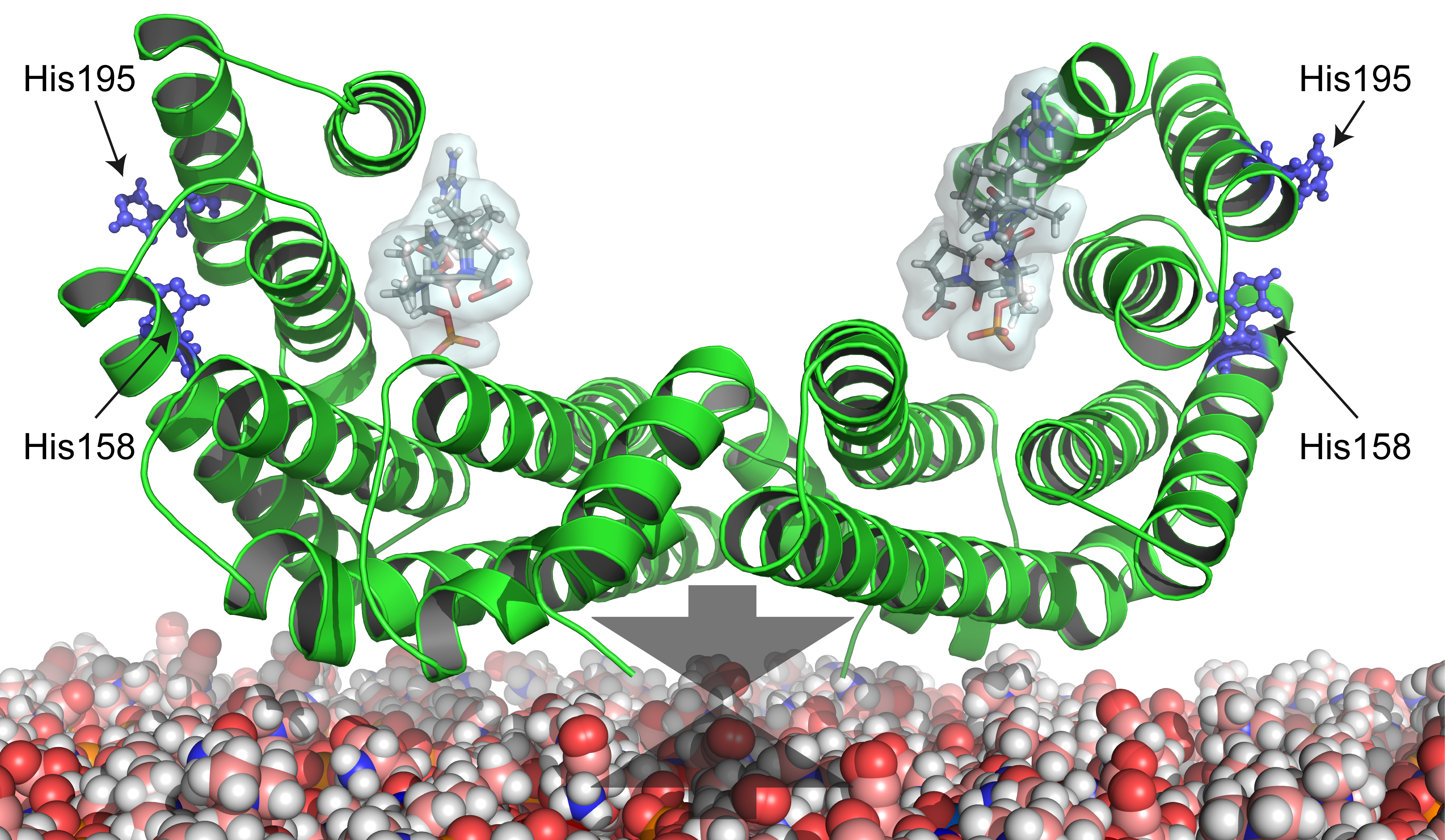

Supplement: Figure S7 — Model of the predicted orientation of ligand-bound 14-3-3γ for optimal interaction with negatively charged membranes. The protein has a positive surface electrostatic potential at the N-terminal dimerization region especially in the presence of bound phosphopeptide (Figure 4), which gives the acidic protein adequate properties for membrane interaction and subsequent intercalation through amphipathic helices A, B and D. The proximity to the membrane also induces the appearance of cationic patches around His158 and His195, aiding to stabilize the membrane bound conformation. (TIF) [file pone.0049671.s007.tif]
